# Using Multiple Sensory Profiling Methods to Gain Insight into Temporal Perceptions of Pea Protein-Based Formulated Foods

**DOI:** 10.3390/foods9080969

**Published:** 2020-07-22

**Authors:** Audrey Cosson, Isabelle Souchon, Julia Richard, Nicolas Descamps, Anne Saint-Eve

**Affiliations:** 1 Univ Paris Saclay, UMR SayFood, AgroParisTech, INRAE, F-78850 Thiverval Grignon, France; audrey.cosson@inrae.fr (A.C.); julia.richard@inrae.fr (J.R.); 2 Roquette Frères, 10 rue haute loge, F-62136 Lestrem, France; nicolas.descamps@roquette.com; 3 Avignon Univ, UMR SQPOV, INRAE, F-84000 Avignon, France; isabelle.souchon@inrae.fr

**Keywords:** formulation, legume, profile, TDS, multi-intake, bitter, beany, astringent

## Abstract

The food industry is focused on creating plant-based foods that incorporate pea protein isolates. However, pea protein isolates are often described as having persistent beany, bitter, and astringent notes that can decrease the desirability of the resulting foods and make static sensory profiling difficult. To obtain more realistic descriptions of the sensory experiences associated with this category of products, researchers should consider using temporal methods and multi-intake methods, which allow consumers to evaluate whole food portions. This study aimed to understand better how product composition affected the sensory perception of pea protein-based beverages using three different sensory profiling methods. Particular focus was placed on beany, bitter, and astringent notes. Twelve pea protein-based beverages were formulated; they varied in pea protein type (pellet vs. isolate) and their content of gellan gum, salt, sunflower oil, sugar, and soy lecithin. They were evaluated by 16 trained panelists using three sensory profiling methods: static block profiling, mono-intake temporal dominance of sensations (TDS) profiling, and multi-intake TDS profiling. The static block and mono-intake TDS profiling methods yielded complementary results about the impact of beverage composition on attribute perceptions. Static block profiling revealed that beaniness was mainly affected by gellan gum and oil content and that bitterness and astringency were mainly affected by protein type and gellan gum content. Mono-intake TDS profiling highlighted the dynamics of beaniness and the strong persistence of astringency, and its results suggested that higher gellan gum and salt contents could limit this persistence. Multi-intake TDS profiling found that, throughout the consumption of a full product portion, beaniness and bitterness decreased, indicating an adaptation effect, while fattiness increased, indicating a build-up effect. This study has increased the understanding of how pea protein-based beverages are perceived under conditions that more closely resemble those associated with real-life consumption. It has also revealed how product formulation can reduce bitterness and astringency.

## 1. Introduction

Consumers are increasingly seeking out healthy, ethically produced, and eco-friendly foods. In this context, plant proteins are proving to be a great success. Yellow field pea (*Pisum sativum L*.) is becoming a common ingredient in plant-based foods [1] because it has a low level of allergenicity and high nutritional value. It also helps ensure the nutritional balance of amino acids in grain-based diets. Yellow field pea isolates also have desirable functional properties: they have excellent emulsification, foaming, gelation, and whipping capacities [2,3]. They are used in the formulation of many types of foods, including dietary supplements, bakery and confectionery products, beverages, yogurts, ice creams, meat products, and alternatives to meat and dairy products.

That said, consumers usually describe pea protein-based foods as having strong beany, bitter, and astringent notes, which can decrease desirability. These attributes have different chemical origins. Indeed, beaniness is the complex flavor perception associated with bean products [4] and results from the complex composition of volatile aroma compounds found in pulses [5]. Bitterness arises from the interaction of bitter compounds (e.g., amino acids, phenolics) with the TAS2R family of receptors, which are found on the apical membranes of taste receptor cells [6,7]. Finally, astringency is produced by “the complex sensations due to shrinking, drawing, or puckering of the epithelium,” and it results from interactions between phenolic compounds and saliva proteins [8,9].

To develop novel products with less pronounced beany, bitter, and astringent notes, food production companies combine pea proteins with several other ingredients (e.g., fat, salt, sugar, flavoring agents, and/or texturizing agents). However, successfully formulating new products from combinations of these ingredients can be challenging and requires a great deal of trial and error. Sensory profiling is a valuable tool in this context: it can be used to explore the impact of food composition on the perceived sensory characteristics of formulated foods, and thus, allows target food products to be obtained more quickly. Many studies have used static block sensory profiling to examine how formulation affects sensory perceptions, the physico-chemical interactions between the different constituents of the food matrix, and the interactions between perceptions of texture, sapidity, and flavor [10,11,12,13,14,15,16,17,18]. 

However, static methods cannot quantify the dynamic mechanisms that play an essential role in how consumers experience foods. Indeed, the oral processing of food includes mastication, salivation, and tongue movements, leading to a complete transformation of food in the mouth. Food transformation has major consequences on food perception and perception persistence [19]. Retronasal aroma perception is affected by interactions among volatile compounds, and the levels of salivary compounds are not constant throughout food consumption [20,21]. Sensations of astringency and bitterness often go hand in hand [22] and slowly develop in the mouth after ingestion. They also increase following repeated exposure [23]. 

Temporal sensory profiling methods are increasingly being used to take these phenomena into account and to obtain a more realistic picture of the sensory experiences elicited by food products. One widely adopted approach is the temporal dominance of sensations (TDS) method. It yields information on the sequence and duration of dominant sensations [24]. The dominant sensations to occur are those that attract the most attention from consumers [25]. The TDS method has been used in tandem with static sensory profiling to evaluate different types of products, making it possible to identify sensory characteristics that are not picked up by one method alone. For example, this combined approach has proven to be useful for studying the persistence of gel containing odorants [26]; interactions between texture and aroma in model candies [27]; solid foods with contrasting textural layers (i.e., fish sticks) [28]; interactions between olive oil composition and pureed beans and tomatoes [29]; the influence of aroma on taste and texture in an apple matrix [30]; and the key flavors perceived in strawberries [31].

Typically, the TDS method is applied to a single instance of food intake (i.e., one bite of solid food or one sip of a beverage). However, in real life, food consumption involves a series of instances of food intake. Several studies have shown that repeated intake of a product can change the perception of product attributes due to sensory adaptation and/or perception persistence [32,33,34]. The multi-intake TDS method can provide a sensory profile for a full portion of food. It has recently proven its utility in studies evaluating the influence of wine on cheese perception [35] and in studies characterizing the sensory properties of an oral nutritional supplement [36], fat-free strawberry yogurts [37], and yogurts with granola [38]. 

This study aimed to understand better how product composition affected the sensory perception of pea protein-based beverages using three different sensory profiling methods. Particular focus was placed on the perception of beany, bitter, and astringent notes. Trained panelists analyzed lab-formulated beverages using three sensory profiling methods: static block profiling, mono-intake TDS profiling, and multi-intake TDS profiling. Analyses were centered on the effects of food composition (protein type, gellan gum content, salt content, and oil content) on texture, sapidity, and aroma, as well as on the sensory interactions of flavor with taste and texture. Finally, the usefulness of a combined sensory profiling approach was discussed.

## 2. Materials and Methods

### 2.1. Materials

Water (Evian, France), gellan gum (Texturas Ferran Adria, Spain), salt (Auchan, France), sunflower oil (Auchan, France), sugar (Daddy, France), soy lecithin (Louis Francois, France), and commercial pea protein isolates were the ingredients used to formulate the beverages. Two Thermomix^®^ TM5^TM^ appliances (Vorwerk, Germany) were employed to standardize product preparation.

### 2.2. Product Preparation

In this study, different pea protein-based beverages were created in the lab. Two mixture designs were used to produce a wide range of plant-beverages from different ingredients while being realistic in terms of ingredient concentrations. The first mixture design was formulated with pea protein isolates and had three independent variables with two levels: sunflower oil concentration (0% or 1.5%), gellan gum concentration (0.12% or 0.5%), and salt concentration (0.08% or 0.12%). The second mixture design was formulated with pea pellets and also had two independent variables: the protein, sunflower oil concentration (0% or 1.5%), and two levels of gellan gum concentration (0.12% or 0.5%). Thus, the total number of trials was 12 (composition and ingredient concentrations are in Table 1). 

First, pea protein pellets were obtained as follows: water and pea protein isolates were slowly mixed together (96% [w/w] water, 4% [w/w] pea protein isolate), and then left to hydrate for 60 min at 4 °C under stirring. The pellet and supernatant were separated via centrifugation at 6000 rpm at 4 °C for 10 min. The pellet was stored at 4 °C for a maximum of 2 h before the beverages were made.

Second, the beverages were created using the following method: the water was mixed and heated (3 min, 50 °C, a speed setting of 2.5) in one of the Thermomix appliances. The sugar, salt, pea protein (isolate or pellet), and gellan gum were then gradually mixed into the water (30 min, 50 °C, a speed setting of 4.5). Simultaneously, the sunflower oil was heated (1 min, 65 °C, speed setting of 1.5) in the second Thermomix. The soy lecithin was then mixed into the sunflower oil (3 min, 65 °C, a speed setting of 2). The contents of the first Thermomix were added to the contents of the second Thermomix and combined without heating (5 min, speed setting of 5). After this step, the overall mixture was heated (6 min, 90 °C, a speed setting of 3.5). Immediately after preparation, the beverages were stored at 4 °C until they were used in the sensory profiling sessions. The Thermomix appliances were cleaned by filling them with a mixture of 2 L of water, 100 mL of white vinegar, and 5 mL of dishwashing liquid, which was then heated (5 min, 70 °C, a speed setting of 1). The appliances were subsequently thoroughly rinsed with hot water and stored at 4 °C until they were used next in order to prevent any bacterial growth.

Rheological tests were performed on each beverage to verify repeatability, and the microbial safety of the products was tested by a certified external laboratory (Eurofins Scientific, France). The products were served to the panelists at room temperature (20 °C) in transparent cups (29.5 mL) identified with three-digit codes.

### 2.3. Experimental Conditions

Sixteen panelists (15 women and 1 man, 18–39 years in age) were recruited based on their desire and availability to participate in a long-term study. Two of the panelists had participated in a study that focused on the sensory characterization of pea protein solutions the year before. The other panelists had no prior experience with pea protein-based products. The panelists were told the overall aim of the experiment. They gave their free and informed consent to participate in the study and received compensation for their participation. They were asked not to eat, drink, or smoke for at least 1 h before the training sessions and evaluation sessions. Panelists performed the sensory evaluations in individual booths under white light in an air-conditioned room (20 °C).

Panelists had to analyze the beverages using three different sensory profiling methods: a static block method [39], a mono-intake TDS method, and a multi-intake TDS (multi-TDS) method. To account for the order in which the beverages were experienced and any potential carry-over effects, beverage order was balanced across panelists using a Latin square. 

A palate-cleansing protocol was used between beverages to reduce sensation build-up: panelists had to consume an apple slice, drink water, and wait for 40 s before consuming the following beverage [39]. As some beverages were viscous, participants were instructed to intake beverages with spoons, instead of sipping for the three profiling methods. 

Sensory analysis was managed using Fizz Acquisition software (v. 2.51, Biosystemes, France).

### 2.4. Attribute Selection and Panelist Training

Panelists were asked to complete a check-all-that-apply (CATA) questionnaire. It listed 30 attributes, and panelists could add more. For the final list and the validation process with the panelists, we retained the attributes that were mentioned most of the time, and that allowed the products to be clearly distinguished. These 11 attributes were salty, bitter, astringent, sweet, fat, pea, almond, nuts, broth, mouthfeel, and overall aromatic intensity (Table 2). As the study was conducted in French, the terms used in French, as well as their translation into English, are presented. 

The panelists were trained to evaluate the intensity of these attributes along an unstructured scale (range: 0–10) using external references. Training took place over 10 sessions that each lasted 45 min. Afterward, panelist performance was evaluated and verified. Overall performance was assessed using ANOVAs with three independent variables (product type, panelist ID, and replicate) and their first-order interactions. There was a product effect, indicating that panelists distinguished among the different beverages (*p* < 0.05). The significance of various interactions revealed whether the panelists consistently scored attributes across replicates (panelist*replicate), whether there was consistency in scoring among panelists (product type*panelist ID), and whether panelists scored products consistently across replicates (product type*replicate). The performance of individual panelists was also evaluated based on their ability to discriminate among beverages and on repeatability criteria. 

### 2.5. Static Block Profiling

**Figure 1:**
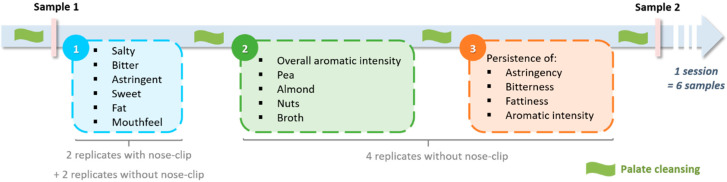
Schematic representation of the static block profiling method used in this study.

Panelists were asked to score the attributes of the 12 beverages using a static block profiling method adapted from the technique used in Cosson et al. [39]. They had to evaluate six different beverages per session and were unaware of beverage identity. They were exposed to four replicates of each product. In total, the panelists evaluated the products over 8 different sensory sessions during four weeks of evaluation. For two replicates, sapidity and texture were evaluated using a nose-clip, and aroma attributes were evaluated without using a nose-clip. For two replicates, all the attributes were evaluated without the nose-clip (Figure 1). The panelists were asked to evaluate attribute intensity as during the training process (along an unstructured scale ranging from 0 to 10). Attributes were assessed in blocks of 4, 5, and 6. First, the panelists had to evaluate sapidity and texture. Second, they had to evaluate aroma. Third, they had to evaluate attribute persistence (using a shorter list of attributes).

### 2.6. Mono-intake Temporal Dominance of Sensations Profiling

Panelists were asked to evaluate the 12 beverages using a mono-intake TDS method—where they evaluated the change in attribute intensity for 120 s after taking a sip of a given beverage. Panelists were exposed to two replicates of each beverage, and they evaluated six beverages per session. In total, the panelists evaluated the products over 4 different sensory sessions during two weeks of evaluation. The attributes were the same as in the static block profiling method except for overall aromatic intensity, which was removed because it was not relevant in this method. Watery was added as an attribute, and it was described to panelists as being the opposite of the fat attribute. Another attribute was also added: “I swallowed.” All the attributes were presented simultaneously on the computer screen. Attribute order was the same for each panelist for all the mono-intake TDS sessions but was randomly assigned and balanced among panelists.

The evaluation process started as soon as the panelists took a sip of the beverage. The panelists then had to click on the attribute that they perceived as dominant, which was defined for them as “the attribute that draws the most attention.” When this dominant attribute changed, the subject had to click on the new dominant attribute. The panelist was free to choose the same dominant attribute several times or, conversely, to never select a dominant attribute. The panelists also had to click on the button “I swallowed” each time they swallowed the beverage or their saliva. 

For each panelist and each beverage, the following data were collected: the time at which an attribute was selected as dominant, the specific attribute, the time that had elapsed before the panelist clicked on “I swallowed” for the first time (i.e., the panelist had largely consumed the product), and the number of times that the panelist clicked on the button “I swallowed.” 

### 2.7. Multi-intake Temporal Dominance of Sensations Profiling

**Figure 2:**
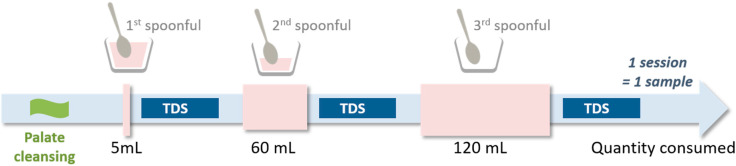
Schematic representation of the multi-intake temporal dominance of sensations (TDS) method used in this study.

Panelists were asked to evaluate two beverages using a multi-intake TDS method. These two beverages were chosen based on the static block profiling results, and the mono-intake TDS profiling results. They contained different protein types (isolate vs. pellet), had a low level of astringency persistence, and displayed different temporal sensory profiles despite having the same gellan gum, salt, and oil contents. The multi-intake TDS profiling method can be used to evaluate changes in attribute perceptions as people consume a full portion of a product (Figure 2). Here, a portion was defined as 120 mL, which is equivalent to an entire ready-to-drink beverage or a serving of yogurt. First, the panelists had to cleanse their palates. Throughout the session, they were not allowed to consume anything except the beverage to allow for the possible cumulative effects of persistent sensations. Second, the panelists evaluated the beverages using the same general approach as in the mono-intake TDS profiling method, except that a given beverage was evaluated at three time points. The first evaluation took place after the first spoonful of the beverage was consumed (hereafter, first spoonful). The second evaluation took place after panelists had consumed 60 mL of the beverage (~half the portion); they then had to evaluate a second spoonful of the beverage (hereafter, second spoonful). The third evaluation took place after panelists had consumed the remaining 60 mL of the beverage, and they then had to evaluate a final spoonful of the beverage (hereafter, third spoonful). Thus, we obtained three sets of data reflecting the shift in sensations from the beginning to the end of beverage consumption. No time limits were placed on this process. Panelists were exposed to two replicates of each product. One replicate of one product was evaluated per session, resulting in a total of four sessions. 

### 2.8. Statistical Analysis

The data were automatically acquired using Fizz Acquisition software (v. 2.51; Biosystemes, 1990). Data analysis was performed using XLSTAT (Addinsoft, 2017, Paris, France) and R (R Core Team, 2019). The threshold for statistical significance was α = 0.05.

The static block profiling data were analyzed using ANOVAs. To assess panelist performance, ANOVAs were carried out in which product type, panelist ID, and replicate were fixed effects, and there were first-order interactions. Post-hoc comparisons were then performed to interpret the specific effect of product type (Newman-Keuls method). To analyze the effect of beverage composition on attribute perception, ANOVAs were performed in which panelist ID, protein type, gellan gum content, salt content, oil content, and nose-clip use were fixed effects, and there were first-order interactions. 

In the case of the mono-intake TDS profiling analyses, the time to the first instance of swallowing and the total duration of the evaluation period were extracted from the data collected during the sessions. ANOVAs were performed in which product type, panelist ID, and replicate were fixed effects, and there were first-order interactions. For the multi-intake TDS profiling data, the ANOVAs had product type, panelist ID, replicate, and spoonful ID as fixed effects and included first-order interactions.

Relative attribute dominance (i.e., the percentage of panelists who perceived a given attribute as dominant) was determined for each beverage at each time point, and the TDS curves were graphed. As suggested by Pineau et al. [24], two lines were drawn on the TDS graph: one line representing the relative dominance an attribute could achieve by chance alone when considering all the attributes evaluated and one line representing the minimum relative dominance an attribute must obtain for the result to be significantly different from that expected by chance alone (binomial distribution, α = 0.05). 

## 3. Results

### 3.1. Panelist Performance

The static block profiling data were used to examine how consistent panelists were in their scoring of attribute intensity (three-way ANOVAs; Table 3). Product type was significant for 15/15 attributes, so the panelists were able to distinguish among the beverages. The interactions between replicate and product type were not significant for 10/15 attributes (except for sweet, mouthfeel, the persistence of bitterness, persistence of fattiness, and persistence of overall aromatic intensity). Replicate was not significant for 12/15 attributes (except for salty, pea, and persistence of overall aromatic intensity), but the interaction between panelist ID and replicate was significant for 11/15 attributes (all except bitter, mouthfeel, pea, and broth). However, in the latter case, the F-values were low compared to the F-values for the product effects. Panelist ID and the interaction between panelist ID and product type were significant for 15/15 attributes. Such interactions are common when sensory attributes are evaluated using unstructured scales, and they are difficult to control even when panelists have undergone extensive training [40,41]. These results nonetheless suggest that the panelists’ scoring was consistent (repeatable and homogeneous) for the majority of attributes. For three attributes (bitter, pea, and almond), there was some inconsistency between panelists, which was taken into account in the analysis of the results.

### 3.2. Impact of Beverage Composition on Perceived Attribute Intensity

**Figure 3:**
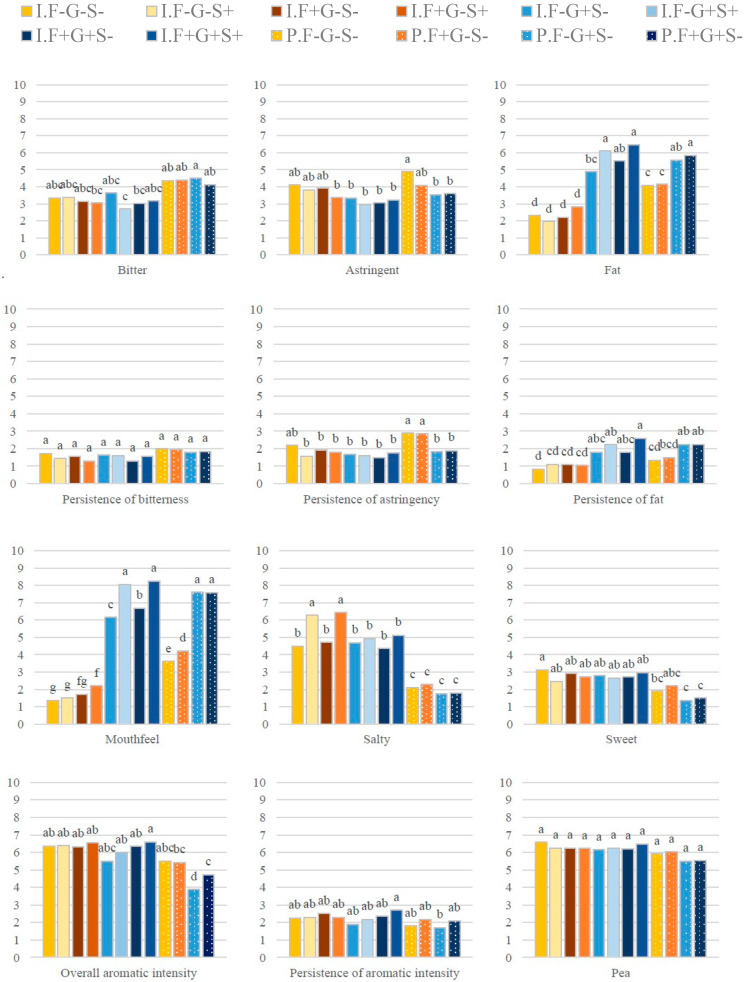

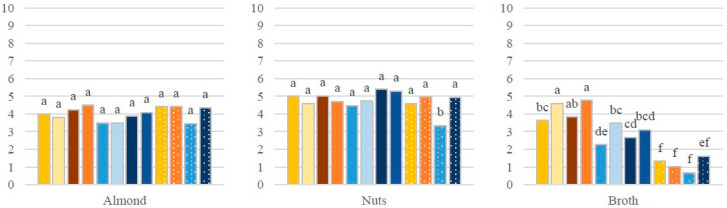
Mean attribute intensities (across replicates and panelists) of the 12 beverages containing different levels of the same ingredients as evaluated using static block profiling (differences in letters indicate significant differences among groups as revealed by the Newman–Keuls post-hoc analysis). Intensity scores could range from 0 to 10. Abbreviations: I = protein from isolate (*solid color*), P = protein from pellet (*dotted color*), F+ = 1.5% oil content (*dark color*), F− = 0% oil content, G+ = 0.5% gellan gum content (*blue*), G- = 0.12% gellan gum content (*orange*), S+ = 0.12% salt content (*light color*), and S− =0.08% salt content.

The static block profiling data were also used to examine the effects of beverage composition on attribute intensity (five-way ANOVAs; Table 4). The mean attribute intensities (across replicates and panelists) for the different beverages and the differences among groups (Newman–Keuls post-hoc analysis) are shown in Figure 3.

Sensory interactions between taste and flavor and between texture and flavor were examined. When panelists were wearing the nose-clip, they perceived the bitter and salty notes as more intense (F = 14.71 and F = 4.17, respectively) than when they were not wearing the nose-clip (4.00 vs. 3.45 and 3.98 vs. 3.70, respectively). 

Protein type influenced the perception of 14/15 attributes (not almond). The most affected attributes were salty (F = 241.07), mouthfeel (F = 233.58), and broth (F = 142.49). Compared to isolate-based beverages, pellet-based beverages were perceived as more bitter and fatty with a more pronounced mouthfeel and more persistent astringency and bitterness; they were also perceived as less salty, sweet, and aromatically intense with less persistent overall aromatic intensity. 

Gellan gum content (0.5% vs. 0.12%) influenced the perception of 9/15 attributes (not bitter, sweet, pea, nuts, the persistence of bitterness, or the persistence of overall aromatic intensity). The most affected attributes were mouthfeel (F = 1769.43) and fat (F = 118.24). Beverages with 0.5% gellan gum content were perceived as fattier with a more pronounced mouthfeel; the persistence of fattiness was also greater. These beverages were also perceived as less salty and astringent with a lower overall aromatic intensity and less persistent astringency. Their almond and broth notes were also less-pronounced.

Salt content (0.08% vs. 0.12%) influenced the perception of 5/15 attributes (salty, fat, mouthfeel, broth, and the persistence of fattiness). Interestingly, the most affected attributes were mouthfeel (F = 82.71) and salty (F = 49.64). Unsurprisingly, beverages with 0.12% salt content were perceived as saltier, and they were also perceived as fattier and brothier with a more pronounced mouthfeel. 

Oil content (1.5% vs. 0%) influenced the perception of 6/15 attributes (fat, mouthfeel, overall aromatic intensity, almond, nuts, and the persistence of overall aromatic intensity). The most affected attribute was the mouthfeel (F = 19.10). Consequently, oil content appeared to have more moderate effects than protein type, gellan gum content, and salt content. Compared to beverages without oil, beverages with oil were perceived as fattier with a more pronounced mouthfeel. They were also perceived as having greater overall aromatic intensity, more persistent overall aromatic intensity, and stronger notes of almond and nuts. 

Except in the case of protein type, beverage composition did not significantly affect the perception of bitterness. Only protein type and gellan gum content influenced the perception of astringency.

There were interactions between protein type and gellan gum content that significantly impacted 5/15 attributes (fat, mouthfeel, overall aromatic intensity, broth, and the persistence of astringency). When a beverage was made with pellet-based protein and contained 0.5% gellan gum, its fattiness and mouthfeel were perceived as more intense, whereas its overall aromatic intensity and brothiness were perceived as less intense. When the gellan gum content was lower (0.12%), the persistence of astringency was perceived as lower. There were also interactions between gellan gum content and salt content, which affected 4/15 attributes (salty, fat, mouthfeel, and the persistence of fattiness). Beverages containing 0.12% gellan gum and 0.12% salt were perceived as saltier and fattier with a more pronounced mouthfeel and more persistent fattiness. The interaction between gellan gum content and oil content significantly impacted 2/15 attributes (nuts and overall aromatic intensity). Beverages containing 0.5% gellan gum and 1.5% oil were perceived as nuttier and as having greater overall aromatic intensity. The other interactions were not significant. 

For the four attributes whose persistence was evaluated (bitter, fat, astringent, and overall aromatic intensity), the mean intensity of attribute persistence was around 2/10, which was lower than the mean intensity of the stand-alone attributes during beverage evaluation. Consequently, static block profiling appears to provide limited information about attribute persistence, at least for the attributes tested. Furthermore, the intensities for the stand-alone attributes (blocks 1 and 2, Figure 1) were correlated strongly with the intensities for attribute persistence (block 3, Figure 1) (R^2^ = 0.84 for astringent and the persistence of astringency; R^2^ = 0.81 for bitter and the persistence of bitterness; R^2^ = 0.95 for fat and the persistence of fattiness; R^2^ = 0.79 for overall aromatic intensity and the persistence of overall aromatic intensity). Thus, temporal sensory profiling is needed to provide better-quality information on attribute persistence.

### 3.3. Results of Mono-intake Temporal Dominance of Sensations Profiling

**Figure 4:**
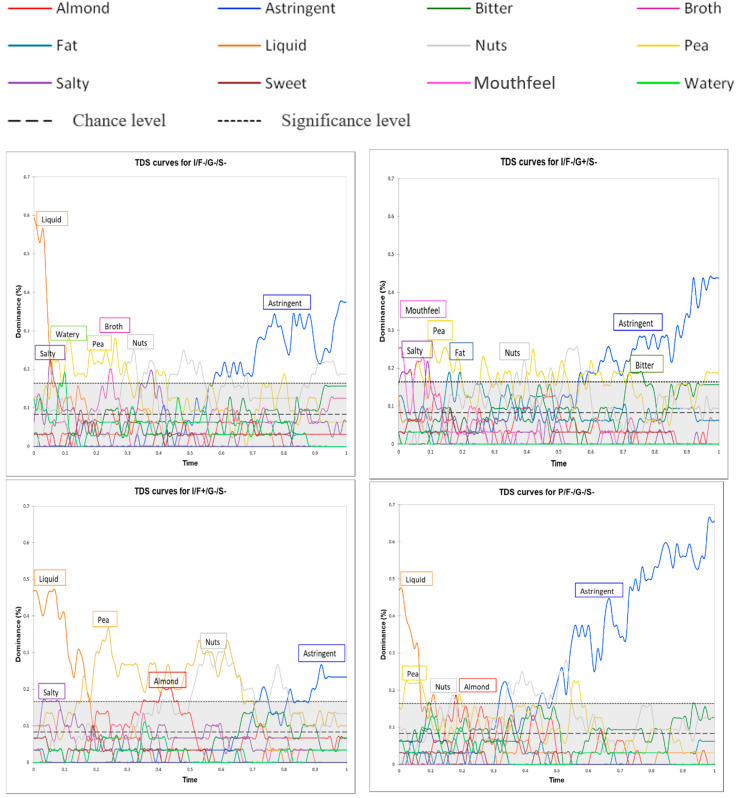

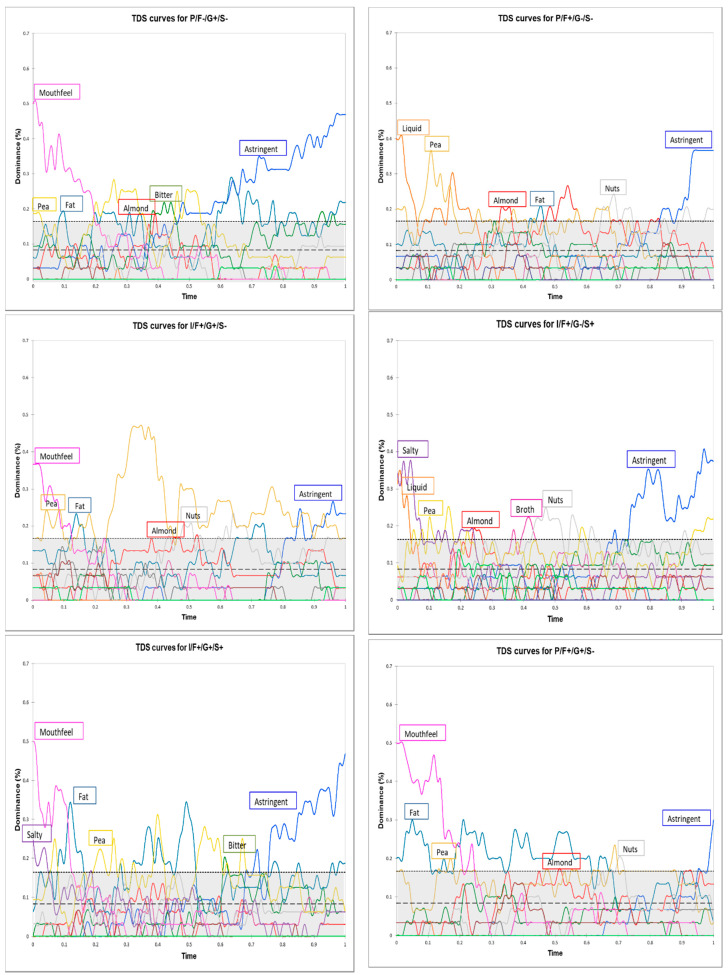

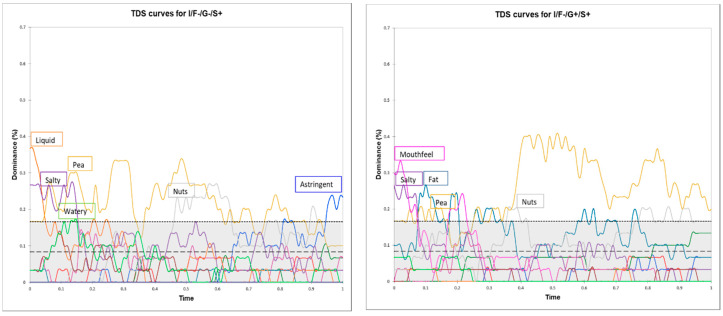
Standardized (mono-intake temporal dominance of sensations) TDS curves for the 12 pea protein-based beverages used in this study. The curves depict attribute dominance over time. The two horizontal lines indicate the relative dominance an attribute could achieve by chance alone (chance level) and the minimum relative dominance an attribute needed to obtain for the result to be significantly different from that expected by chance alone (significance level). Abbreviations: I = isolate, P = pellet, F+ = 1.5% oil, F− = 0% oil, G+ = 0.5% gellan gum, G- = 0.12% gellan gum, S+ = 0.12% salt, and S− = 0.08% salt.

The perceived dominant attributes of the beverages across the consumption period can be seen in Figure 4.

The first instance of swallowing is not indicated because it always occurred at the very beginning of the evaluation period (within 4.32–7.90 s of starting the 120-s period), which underscores the effect of the aftertaste on attribute dominance. Beverage composition affected the time to the first instance of swallowing and total evaluation duration (three-way ANOVAs; Table 5). Differences in both these dependent variables (F = 3.43 and F = 6.51, respectively) were observed among beverages. Beverages with the least pronounced mouthfeel were swallowed the fastest (I/F−/G−/S−, I/F+/G−/S+, I/F−/G−/S+, and I/F+/G−/S− were first swallowed within 4.32–4.93 s). The beverage with the most pronounced mouthfeel was swallowed the slowest (I/F+/G+/S+ product was first swallowed within 7.90 s), and its evaluation duration was the longest. There were also marked differences among panelists in both variables (time to first swallow: range: 0–42.25 s, mean: 30.48 ± 5.11 s, and F=35.82; evaluation duration: range: 10.75–120 s, mean: 24.49 ± 5.90 s, and F = 158.70). The interactions between product type and panelist ID were also significant (time to first swallow: F = 1.40 and evaluation duration: F = 1.98). The pronounced variability in both variables reflected the prominent differences in food oral processing among panelists. 

During the evaluation period, panelists described the 12 beverages using at least five attributes. Specific sensory phases were also identified. In the first part of the evaluation period, for all beverages, the dominant attributes were those associated with texture and sapidity (liquid, mouthfeel, and salty). Then, depending on the specific beverage, the attributes related to aroma (almond, pea, and broth), texture (fat, watery), and sapidity (salty, bitter) were simultaneously dominant. Finally, in the last part of the evaluation period, astringency was dominant for all the beverages. 

When the beverages were examined separately, the results were consistent with those obtained using static block profiling, as illustrated by the high RV coefficient of 0.796 between static and TDS profiling data (multiple factor analysis). For the pellet-based beverages versus the isolate-based beverages, the attributes fat, mouthfeel, and astringent remained dominant for a longer period, while the attributes salty, almond, pea, and broth remained dominant for a shorter period. The dominance of the attributes fat and mouthfeel lasted longer in beverages containing 0.5% gellan gum than in beverages containing 0.12% gellan gum. Unsurprisingly, the dominance of the attribute salty lasted longer in beverages containing 0.12% salt than in beverages containing 0.08% salt. Similarly, the dominance of the attribute fat persisted for longer in the beverages containing oil (1.5%) than in the beverages without any oil. 

However, mono-intake TDS results also provided additional information, notably with regards to bitterness and astringency. Panelists seemed to barely perceive astringency in the beverages containing 0% oil and 0.12% salt (I/F−/G−/S+ and I/F−/G+/S+). This attribute was also much less dominant in beverages containing 1.5% oil and 0.08% salt (P/F+/G+/S−, I/F+/G+/S−, I/F+/G−/S−, and P/F+/G−/S−). The attribute bitter was rarely perceived as dominant, and when it was, it was only in the three beverages containing the higher percentage (0.5%) of gellan gum (P/F−/G+/S−, I/F−/G+/S−, and I/F+/G+/S+).

Based on these results, two beverages (I/F+/G+/S− and P/F+/G+/S−) were selected for evaluation with the multi-intake TDS method because they displayed weakly persistent astringency and different temporal profiles for the attribute pea (a contributor to beaniness). 

### 3.4. Results of Multi-intake Temporal Dominance of Sensations Profiling

In the multi-intake TDS method, panelists had to evaluate attribute dominance at three time points. Once after consuming the first spoonful of beverage, once after consuming 60 mL (half) of the beverage, and once after consuming 120 mL (all) of the beverage. 

Product type affected the time to the first instance of swallowing and total evaluation duration (four-way ANOVAs; Table 6). Beverages differed in the time to the first swallow (F = 4.70). The beverage with the less pronounced mouthfeel was swallowed faster (I/F+/G+/S−: 5.87 s) than the beverage with the more pronounced mouthfeel (P/F+/G+/S−: 6.73 s). There were differences in both variables among the evaluation time points (time to first swallow: F = 11.48 and evaluation duration: F = 7.10). Time to the first swallow was longest after the first spoonful, regardless of product type (1st spoonful: 7.55 s; second spoonful: 6.12 s; third spoonful: 5.23 s), as was the length of the evaluation period (1^st^ spoonful: 48.11 s; second spoonful: 44.39 s; and third spoonful: 41.82 s). These results likely reflect panelist fatigue and adaptation effects.

**Figure 5:**
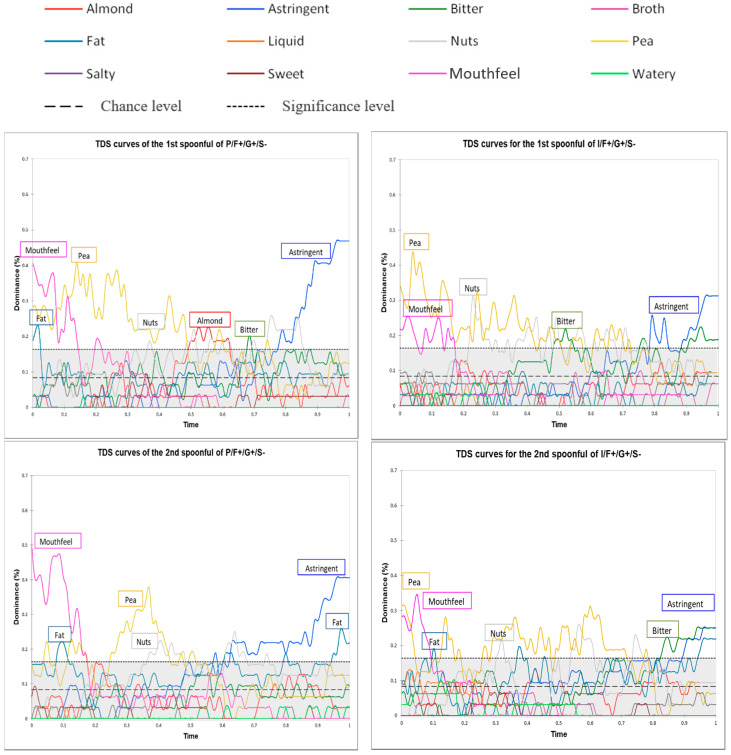

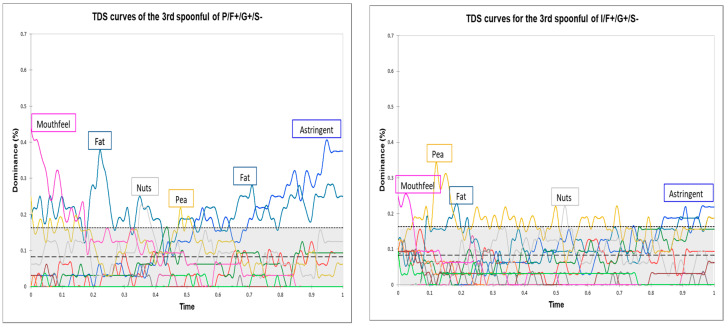
Standardized TDS curves for the two pea protein-based beverages (I/F+/G+/S− and P/F+/G+/S) evaluated using multi-intake TDS profiling. The curves depict attribute dominance over time (i.e., following the first spoonful, the second spoonful [after consuming 60 mL], and the third spoonful [after consuming 120 mL]). The two horizontal lines indicate the chance level and the significance level (see Figure 4). Abbreviations: I = isolate, P = pellet, F+ = 1.5% oil, G+ = 0.5% gellan gum, and S− = 0.08% salt.

Attribute dominance over time for the two beverages is shown in Figure 5. These two beverages were selected from the evaluation with the multi-intake TDS method because they displayed weakly persistent astringency and different temporal profiles for the attribute pea. As in the results for the mono-intake TDS method, panelists described the beverages as having at least five different attributes. The sequence of dominant attributes was also similar. In the first part of the evaluation period, the dominant attributes for I/F+/G+/S− were mouthfeel and pea; for P/F+/G+/S−, they were mouthfeel and fat. Then, the attributes of fat, pea, nuts, and almond were more dominant, but their relative ranks were dependent on product type and spoonful ID. In the last part of the evaluation period, the attributes astringent and bitter were dominant for I/F+/G+/S−, and the attributes astringent and fat were dominant for P/F+/G+/S−.

The results for the first spoonfuls consumed during the multi-intake TDS sessions did not fully match the results for the single spoonfuls consumed during the mono-intake TDS sessions. When I/F+/G+/S− was evaluated using the multi-intake TDS method, the attributes bitter and nuts were dominant for the longest amount of time after the first spoonful of beverage was consumed. In contrast, when the mono-intake TDS method was used, the attributes mouthfeel, fat, and pea were the most dominant. Similarly, when P/F+/G+/S− was evaluated using the multi-intake TDS method, the attributes bitter, astringent, pea, and almond were dominant for the longest amount of time after the first spoonful of beverage was consumed. In contrast, when the mono-intake TDS method was used, the attributes mouthfeel and fat were the most dominant. These contrasting results may stem from methodological differences. During the mono-intake TDS sessions, panelists evaluated a total of 12 spoonfuls of beverage at random points during a given session. Thus, these single spoonfuls do not truly correspond to the “real” first spoonfuls taken during the multi-intake TDS sessions.

Attributes decreased in dominance throughout the evaluation period for I/F+/G+/S−. Panelists perceived the beverage’s attributes quite differently by the time they reached the end of consumption. For example, the dominance of the attributes pea and astringent declined between the first and the third spoonful (from 45% to 35% and from 32% to 25%, respectively). For P/F+/G+/S−, the same decline in dominance was observed for the attributes pea, nuts, and almond. However, astringency was still highly dominant at the end of the evaluation period, and the attribute fat increased in dominance over time.

## 4. Discussion

This study aimed to understand better how product composition affected the sensory perception of pea protein-based beverages using three different sensory profiling methods. The first part of the discussion focuses on how beverage composition affected the perception of texture and sapidity. The second part examines the perception of aroma and the sensory interactions of flavor with taste and texture. The third part addresses the importance of employing a combination of sensory profiling methods (static/temporal, mono-intake/multi-intake) when evaluating potential food products. 

### 4.1. Perception of Texture and Sapidity

In this study, the composition of the pea protein-based beverages greatly impacted perceptions of texture and sapidity. When the static block profiling method was used (i.e., when sensory attributes were evaluated immediately after consumption), gellan gum content, salt content, and oil content were found to increase the perceived intensity of fattiness and mouthfeel significantly. This result suggests a relationship exists between the two attributes. Similarly, when the mono-intake TDS method was used (i.e., where sensory attributes were evaluated over a 2-min period following consumption), the attribute mouthfeel was perceived as more dominant for beverages with low gellan gum and salt contents. The attribute of fat was perceived as more dominant for beverages with high gellan gum contents that also contained oil. 

When beverages had a lower salt content, the perceived intensity of saltiness was lower (as measured via static block profiling), and the attribute salt was less dominant (as measured via mono-intake TDS profiling). When beverages had higher gellan gum content, the perceived intensity of astringency was lower (as measured via static block profiling), but the attribute bitter was highly dominant (as measured via mono-intake TDS profiling). Here, however, in contrast to other studies, there was no significant effect of fat content on bitterness [42], perhaps because the differences in oil content were small (1.5% vs. 0%).

The type of protein used to make the beverage (isolate vs. pellet) also affected perceptions of texture and sapidity. Based on static block profiling, pellet-based products were perceived as being fattier, bitterer, and less salty and as having a more pronounced mouthfeel. Based on mono-intake TDS profiling, astringency was highly dominant in pellet-based products. Protein type has a compositional effect on food products. Although pea pellets and isolates both contain similar levels of total proteins, pellets are richer in insoluble proteins, while isolates are richer in minerals, sugars, polyphenols, volatile molecules, and peptides. Analyses of protein extracts have identified the proteins and peptides responsible for bitterness: they have hydrophobic side chains rich in proline and leucine [43,44]. Astringency results from the saliva proteins (e.g., salivary amylase, mucin, esterase) binding with the polyphenols present in pea protein isolates, and then precipitating [9,45,46]. Thus, it can be assumed here that differences in protein type were at the origin of differences in attribute perception.

As observed in previous studies, texture attributes initially dominate food perception [24,27,47]. In addition, swallowing occurs more quickly, after a few seconds (during the first part of the evaluation period), for liquid products, a result that could be explained by the oral processing dynamics of liquid foods [48]. While solids need to be fragmented and mixed with saliva to form a cohesive bolus, liquids can be swallowed immediately after being diluted by saliva and warmed to body temperature [49]. Thus, liquids usually remain in the mouth for a much shorter period than do solids. 

The results obtained with multiple-intake TDS profiling (i.e., where the sensory attributes of a full beverage portion were evaluated) revealed a gradual decrease in the dominance of texture attributes and bitterness over time. This decrease was more pronounced for the pellet-based beverage than the isolate-based beverage. Such attributes might become less noticeable after repeated tasting due to sensory adaptation [33]. There was also a gradual increase in perceived fattiness across time, which could be due to the lingering and build-up of sensations [32,33]. These results fit with those from several other studies showing that perceptions of fattiness build up in the mouth due to fat lingering on oral surfaces (i.e., the tongue and the palate) [50,51]. The persistence of the sensation of fattiness may stem from the presence of residual fat or oil in the oral cavity after swallowing, which can increase the attribute’s intensity throughout repeated ingestion [50,52]. 

### 4.2. Perception of Aroma and the Interactions of Flavor with Taste and Texture

Beverage composition greatly influenced the perception of aroma. Static block profiling showed that products with greater gellan gum content were perceived as having lower overall aromatic intensity and less pronounced almond and broth notes. In contrast, mono-intake TDS profiling revealed that the attribute pea was relatively dominant in this beverage type. The impacts of hydrocolloid solutions on the sensory perception of food depend on a large number of variables (e.g., hydrocolloid type, range of viscosity, food matrix type, choice of sensory evaluation technique). Only a few studies have explored the effects of hydrocolloids on the perception of thickened beverages [53,54,55], and, to our knowledge, none have looked at gellan gum. However, these studies generally found that an increase in beverage viscosity led to a decrease in aroma perception [56,57,58], which is consistent with the results of this study.

Beverages containing oil were perceived as having higher overall aromatic intensity and more intense almond and nut notes based on static block profiling. When mono-intake TDS profiling was used, these beverages displayed the highest dominance of almond and the lowest dominance of broth. Past research has repeatedly shown that lipids can modify the sensory perception of food. They function as reservoirs for numerous aroma compounds, resulting in delayed release and perception [59,60,61,62]. In addition, in static block profiling, beverages with a higher salt content were perceived as displaying more intense brothiness, and in mono-intake TDS profiling, they were perceived as having the least dominant almond note. This result can be explained by sodium chloride, causing the salting out of hydrophobic aroma compounds [62].

Protein type influenced the perception of overall aromatic intensity. Pellet-based products were perceived as less aromatic than isolate-based products, based on static block profiling. The results for mono-intake TDS profiling provided additional support for this finding, where the attributes pea and nuts were perceived as less dominant in pellet-based products than in isolate-based products. Previous research has extensively examined interactions in protein-based foods between aroma compounds and proteins [63,64]. These interactions can be modified by different factors: protein conformation and composition; the properties of aroma compounds, such as hydrophobicity; and environmental conditions, such as pH [64,65,66,67]. Thus, it can be assumed that the above sensory differences arose from differences in protein type and, more specifically, differences in interactions between aroma compounds and proteins.

Here, it was found that aroma attributes were dominant during the latter part of the evaluation period, based on mono-intake TDS profiling. This finding concurs with what has been seen in previous studies. During the swallowing process, the liquid bolus is held first on the upper surface of the tongue [68]. During this step, the soft palate is most often closed, and aroma compounds have limited access to the nasal cavity, which may explain why only texture and sapidity attributes were dominant during the initial part of the evaluation period. Then, the tongue generates a wave of pressure that squeezes the liquid backward through the mouth and pharynx toward the esophagus [69]. Immediately after the liquid passes the epiglottis, the soft palate is re-opened [70]. For liquid foods, this is the first moment in which aroma compounds have access to the nasal cavity [71], and the highest aroma release signal is generally observed during the first expiration after swallowing (called the swallow breath) [72]. This series of events may explain why aroma attributes were more dominant during the latter part of the evaluation period. After a few seconds, the concentration of volatile compounds in the mouth and nasal cavity decrease significantly [73]. In contrast, non-volatile compounds remain on oral surfaces (i.e., the tongue and palate) and continue to influence perceptions [50,51], which may explain why astringency was dominant later in the evaluation period. Multiple-intake TDS profiling showed that beaniness gradually decreased over time. This decrease was more-pronounced for pellet-based beverages. However, these attributes might become less noticeable after repeated tasting due to sensory adaptation [33]. 

Beverage composition had a limited effect on the sensory interactions of flavor with taste and texture. However, there were some prominent taste–flavor interactions. When the panelists used nose-clips to evaluate attributes related to texture and sapidity, bitter and salty notes were perceived as less intense than when the nose-clip was not used. Beverages were also perceived simultaneously as more beany, bitter, and salty, suggesting congruent effects. These results are consistent with those found in other studies on bitter beverages. For example, cocoa flavoring enhanced bitterness in a cocoa beverage [74], and the addition of aroma compounds increased bitterness in beers [75]. These results suggest that the effects of congruency induce interactions between taste and aroma.

### 4.3. The Importance of Employing a Combination of Sensory Profiling Methods

Static block profiling, in which beverage attributes were evaluated immediately after consumption, revealed that the perception of beaniness was strongly affected by beverage composition. At the same time, the differences between the different attributes contributing to beaniness (pea, nuts, almond, and broth) were not very pronounced. Mono-intake TDS profiling, in which beverage attributes were evaluated over a 2-min period following consumption, provided more detailed information about differences among beverages, especially in terms of the different attributes contributing to beaniness. In particular, results suggest that pellet-based beverages were perceived as more brothy and less pea-like than isolate-based beverages. Static block profiling found that perceived astringency was moderate, and the intensities for the stand-alone attributes (blocks 1 and 2, Figure 1) were correlated strongly with the intensities for attribute persistence. In contrast, mono-intake TDS profiling highlighted that perceived astringency was strongly persistent over the evaluation period and that the perception of other attributes shifted. The static block profiling method made it possible to rapidly and independently evaluate attribute intensity. However, it is difficult for panelists to assess attribute dominance and intensity at the same time during TDS [19], and thus, there is a risk of interdependence among attributes [27,76]. That said, static block profiling requires panelists to integrate their changing sensory perceptions throughout oral processing to come up with a summary evaluation [77], and it is hard to control the point in the oral process at which products are evaluated. Thus, it makes sense to jointly use static block profiling and TDS profiling to obtain a better understanding of attribute intensity and dominance in food products.

Conventionally, in TDS profiling, different attribute families (taste, texture, and aroma) can be evaluated during different parts of a study ([37]). Here, however, the choice was made to evaluate the different attribute families at once. Although the influence of listing attributes from different families in the same list remains unknown [19], this methodological approach makes it possible to assess all the attributes simultaneously and to identify specific sensory phases. Texture attributes dominated the first part of the evaluation period. Then, depending on the product and the panelist, different attributes became dominant. Finally, in the latter part of the evaluation period, astringency became dominant. 

The results obtained with the multi-intake TDS profiling method underscore that quantifying sensory experiences over time could provide additional information about how consumers perceive foods. For example, perceived fattiness became more dominant over the course of consumption, while other attributes (except astringency) became less dominant, perhaps because repeated tasting led to sensory adaptation. Previous research using multi-intake TDS profiling found that attributes related to texture and sapidity gradually increased over time but that there was no intake effect on how long aroma attributes remained dominant [34,36,37,38,78,79]. However, in these studies, panelists evaluated multiple spoonfuls of product in a row. In contrast, the present study had panelists evaluate spoonfuls of beverage at three distinct periods, corresponding to the beginning, the middle, and the end of the consumption of a full product portion. Another study that examined temporal changes in attribute perceptions during the consumption of an entire portion of an oral nutritional supplement found that there were differences in the aroma attribute “praline” over time [36]. 

These findings raise questions regarding the ideal number of spoonfuls and the amount of product that should be consumed by panelists. Here, it seemed to be more useful to have panelists evaluate spoonfuls taken at specific moments during the consumption of a full beverage portion than to have panelists consume several spoonfuls of beverage in a row. In other contexts, it could make more sense to evaluate multiple spoonfuls consumed ad libitum, such as when the goal is to investigate the effect of sensory-specific satiety, which is a decrease in attribute perception for a specific food following repeated exposure [80]. Nevertheless, both these methodologies (i.e., consumption of a full portion or ad libitum consumption) share the disadvantage that only one replicate of one product can be evaluated per session. Thus, in addition to being time-consuming, there is a risk of failing to pick up on differences among products. For this reason, it is important to explore how spoonful numbers and the amount of product consumed influence the results obtained.

Finally, for food production companies, improving methods for characterizing the sensory profiles of products is key to better understanding consumers’ experiences. This study did not take into account temporal hedonic profiles. However, it could be interesting to combine descriptive and hedonic analyses with multi-intake TDS profiling. This approach could provide further insight into pea protein-based products, leading to their improvement ([36,38]).

## 5. Conclusions and Perspectives

In conclusion, this study’s use of three methods—static block profiling, mono-intake TDS profiling, and multi-intake TDS profiling—helped clarify how the composition of pea protein-based beverages affected sensory perceptions. The static block profiling method, in which beverage attributes were evaluated immediately after consumption, revealed that the perception of beaniness depended on protein type, where it was higher when the pea protein source was an isolate than when it was a pellet. Perceived beaniness also increased when gellan gum content was lower, and the oil content was higher. The mono-intake TDS profiling method, in which beverage attributes were evaluated over a 2-min period following consumption, showed that beverages differed markedly in the dynamics of their aroma attributes. In particular, almond notes were more dominant, and pea notes were less dominant in pellet-based beverages than in isolate-based beverages. These characteristics were accentuated from one spoonful to the next. Perceptions of astringency and bitterness were impacted mainly by protein type and gellan gum content. While static block profiling found a moderate level of perceived astringency, mono-intake TDS profiling highlighted that astringency was strongly persistent and that this persistence seemed to be limited by gellan gum and salt contents. The use of the nose-clip during static block profiling indicated that there were few interactions of flavor with texture and taste. It also yielded evidence of a weak effect of congruency between the bitter/salty notes and the beany note. Specific sensory phases were also identifie: texture attributes were more prominent during initial consumption, and astringency was more prominent during later consumption. Finally, the multi-intake TDS profiling results suggest that, over time, the perception of fattiness built up, and the perception of beaniness shifted because of sensory adaptation. Thus, taken together, this study’s findings have enhanced understanding of sensory perceptions of pea protein-based beverages under conditions that more closely resemble those associated with real-life consumption. They also provide clues for reformulating pea protein-based products to reduce beaniness, bitterness, and astringency.

## Figures and Tables

**Table 1:** Composition (ingredient concentrations [w/w %]) of the pea protein-based beverages used in this study. Abbreviations: I = isolate, P = pellet, F+ = 1.5% oil, F− = 0% oil, G+ = 0.5% gellan gum, G− = 0.12% gellan gum, S+ = 0.12% salt, and S− = 0.08% salt.

Product Name	Protein Type	Sunflower Oil (%)	Soy Lecithin (%)	Gellan Gum (%)	Salt (%)	Sugar (%)	Pea Protein (%)	Water (%)
	(*P or I*)	(*F+ or F−*)		(*G+ or G−*)	(*S+ or S−*)			
I/F−/G−/S−	Isolate	**0.00**	**0.00**	0.12	0.08	1.00	7.00	91.80
I/F−/G−/S+	Isolate	**0.00**	**0.00**	0.12	**0.12**	1.00	7.00	91.76
I/F−/G+/S−	Isolate	**0.00**	**0.00**	**0.50**	0.08	1.00	7.00	91.42
I/F−/G+/S+	Isolate	**0.00**	**0.00**	**0.50**	**0.12**	1.00	7.00	91.38
I/F+/G−/S−	Isolate	1.50	0.10	0.12	0.08	1.00	7.00	90.20
I/F+/G−/S+	Isolate	1.50	0.10	0.12	**0.12**	1.00	7.00	90.16
I/F+/G+/S−	Isolate	1.50	0.10	**0.50**	0.08	1.00	7.00	89.82
I/F+/G+/S+	Isolate	1.50	0.10	**0.50**	**0.12**	1.00	7.00	89.78
P/F−/G−/S−	**Pellet**	**0.00**	**0.00**	0.12	0.08	1.00	7.00	91.80
P/F−/G+/S−	**Pellet**	**0.00**	**0.00**	**0.50**	0.08	1.00	7.00	91.42
P/F+/G−/S−	**Pellet**	1.50	0.10	0.12	0.08	1.00	7.00	90.20
P/F+/G+/S−	**Pellet**	1.50	0.10	**0.50**	0.08	1.00	7.00	89.82

**Table 2:** Definition of the sensory attributes evaluated by the panelists.

Attributes	Attributes in French	Definition
Salty	*Salé*	A fundamental taste—sodium chloride is a typical example
Bitter	*Amer*	The fundamental taste associated with a caffeine solution
Astringent	*Astringent*	A sensation of drying out, roughening, and/or puckering that is felt in the mouth, like when consuming red wine or unripe fruit
Sweet	*Sucré*	A fundamental taste—sucrose is a typical example
Fat	*Gras*	Property relative to the perception of the quantity of fat in the product
Mouthfeel	*Epais*	The way a food feels in the mouth in relation to its viscosity
Overall aromatic intensity	*Intensité aromatique globale*	Total aroma impressions created by the product in the mouth
Pea	*Pois*	The flavor characteristic of beans and bean-based foods
Almond	*Amande*	The flavor associated with almonds
Nuts	*Noix*	The flavor associated with nuts, like walnuts or hazelnuts
Broth	*Bouillon*	The flavor associated with boiled vegetables, soup, or stock

**Table 3:** Results of the three-way ANOVAs (panelist ID, replicate, and product type as fixed effects + their first-order interactions) examining consistency in panelist performance (total degrees of freedom: 739; residual degrees of freedom: 681). Significant *p*-values are in bold (α = 0.05). Abbreviations: Astringent-P = persistence of astringency; Bitter-P = persistence of bitterness; Fat-P = persistence of fattiness; and Aromatic intensity-P = persistence of overall aromatic intensity.

	Panelist ID		Replicate		Product Type		Panelist ID * Replicate		Panelist ID * Product Type		Replicate * Product Type	
	F	*p*-Value	F	*p*-Value	F	*p*-Value	F	*p*-Value	F	*p*-Value	F	*p*-Value
**Salty**	29.47	**<0.01**	5.71	**0.02**	61.90	**<0.01**	4.42	**<0.01**	1.78	**<0.01**	1.34	0.21
**Bitter**	34.83	**<0.01**	0.01	0.92	8.44	**<0.01**	1.22	0.25	2.49	**<0.01**	0.43	0.93
**Astringent**	26.94	**<0.01**	0.95	0.33	5.71	**<0.01**	3.23	**<0.01**	1.78	**<0.01**	0.25	0.99
**Sweet**	23.09	**<0.01**	0.92	0.34	8.14	**<0.01**	2.60	**<0.01**	1.64	**<0.01**	2.03	**0.03**
**Fat**	10.11	**<0.01**	0.49	0.49	62.77	**<0.01**	1.98	**0.02**	2.38	**<0.01**	0.91	0.53
**Mouthfeel**	13.79	**<0.01**	1.07	0.30	358.24	**<0.01**	1.58	0.07	2.00	**<0.01**	3.77	**<0.01**
**Overall aromatic intensity**	9.17	**<0.01**	0.17	0.68	14.71	**< 0.01**	2.55	**< 0.01**	1.93	**< 0.01**	0.87	0.57
**Pea**	29.85	**<0.01**	4.82	**0.03**	2.44	**0.01**	1.48	0.11	2.47	**<0.01**	1.03	0.42
**Almond**	32.78	**<0.01**	0.16	0.69	2.57	**<0.01**	2.61	**<0.01**	1.60	**<0.01**	0.29	0.98
**Nuts**	27.21	**<0.01**	3.69	0.06	5.72	**<0.01**	2.84	**<0.01**	2.60	**<0.01**	0.84	0.59
**Broth**	25.34	**<0.01**	0.04	0.83	41.96	**<0.01**	1.01	0.45	1.76	**<0.01**	0.21	1.00
**Astringent-P**	52.86	**<0.01**	1.41	0.23	9.47	**<0.01**	3.54	**<0.01**	1.86	**<0.01**	1.38	0.19
**Bitter-P**	34.61	**<0.01**	0.02	0.89	2.88	**<0.01**	2.93	**<0.01**	1.71	**<0.01**	1.91	**0.04**
**Fat-P**	79.46	**<0.01**	3.19	0.07	26.28	**<0.01**	1.90	**0.02**	2.89	**<0.01**	1.90	**0.04**
**Aromatic intensity-P**	57.42	**<0.01**	11.22	**0.00**	4.71	**<0.01**	1.90	**0.02**	1.35	**0.01**	1.97	**0.03**

* corresponds to the interaction between replicate and product type.

**Table 4:** Results of the five-way ANOVAs (panelist ID, nose-clip use, oil content, gellan gum content, salt content, and protein type as fixed effects + their first-order interactions) examining the effects of beverage composition on attribute perception using the static block profiling data (total degrees of freedom: 739; residual degrees of freedom: 716). Significant *p*-values are in bold (α = 0.05). Abbreviations: Astringent-P = persistence of astringency; Bitter-P = persistence of bitterness; Fat-P = persistence of fattiness; and Aromatic intensity-P = persistence of overall aromatic intensity.

	Panelist ID		Nose-clip Use		Oil Content		Gellan Gum Content		Salt Content		Protein Type		Oil * Gellan Gum		Gellan gum * Salt		Gellan gum * Protein Type	
	*F*	*p-*Value	*F*	*p-*Value	*F*	*p-*Value	*F*	*p-*Value	*F*	*p-*Value	*F*	*p-*Value	*F*	*p-*Value	*F*	*p-*Value	*F*	*p-*Value
**Salty**	25.39	**<0.01**	4.17	**0.04**	0.24	0.62	27.96	**<0.01**	49.64	**<0.01**	241,07	**<0.01**	0,73	0,39	15,56	**<0.01**	1,15	0,28
**Bitter**	26.64	**<0.01**	14.71	**<0.01**	1.30	0.26	0.65	0.42	1.69	0.19	34.38	**<0.01**	0.00	0.99	1.20	0.27	0.11	0.74
**Astringent**	22.23	**<0.01**	0.86	0.35	2.39	0.12	14.33	**<0.01**	2.00	0.16	5.32	**0.02**	2.76	0.10	0.88	0.35	0.05	0.83
**Sweet**	21.44	**<0.01**	0.88	0.35	1.24	0.27	1.73	0.19	1.36	0.24	55.21	**<0.01**	0.00	0.95	2.89	0.09	1.09	0.30
**Fat**	7.71	**<0.01**	2.53	0.11	6.45	**0.01**	247.75	**<0.01**	13.13	**0.00**	46.78	**<0.01**	0.33	0.57	8.18	**<0.01**	14.18	**<0.01**
**Mouthfeel**	11.39	**<0.01**	0.52	0.47	19.10	**<0.01**	1769.43	**<0.01**	82.71	**<0.01**	233.58	**<0.01**	2.03	0.15	39.03	**<0.01**	23.56	**<0.01**
**Overall aromatic intensity**	7.24	**<0.01**	3.07	0.08	7.39	**0.01**	15.19	**0.00**	2.07	0.15	55.03	**<0.01**	6.78	**0.01**	0.41	0.52	5.41	**0.02**
**Pea**	23.19	**<0.01**	1.95	0.16	0.01	0.94	0.90	0.34	0.00	0.99	10.48	**<0.01**	0.36	0.55	0.98	0.32	0.47	0.49
**Almond**	29.31	**<0.01**	1.67	0.20	9.88	**<0.01**	6.57	**0.01**	0.14	0.71	1.71	0.19	0.98	0.32	0.02	0.88	0.17	0.68
**Nuts**	20.15	**<0.01**	1.86	0.17	12.40	**<0.01**	0.99	0.32	0.69	0.41	8.99	**<0.01**	6.09	**0.01**	1.10	0.29	3.41	0.07
**Broth**	23.17	**<0.01**	0.82	0.36	1.10	0.29	21.10	**<0.01**	31.06	**<0.01**	142.49	**<0.01**	0.65	0.42	0.16	0.69	11.41	**0.00**
**Astringent-P**	39.99	**<0.01**	0.68	0.41	0.03	0.87	16.27	**<0.01**	1.36	0.24	17.26	**<0.01**	0.00	0.97	3.59	0.06	5.01	**0.03**
**Bitter-P**	29.24	**<0.01**	0.04	0.84	2.47	0.12	0.06	0.80	0.83	0.36	8.16	**<0.01**	0.08	0.78	3.07	0.08	0.01	0.90
**Fat-P**	54.04	**<0.01**	3.77	0.05	1.91	0.17	118.24	**<0.01**	14.35	**<0.01**	21.24	**<0.01**	0.00	0.96	6.11	**0.01**	0.02	0.89
**Aromatic intensity-P**	51.62	**<0.01**	2.82	0.09	16.59	**<0.01**	0.15	0.70	1.25	0.26	9.73	**<0.01**	2.12	0.15	4.00	0.05	0.52	0.47

* corresponds to the interaction between replicate and product type.

**Table 5:** Results of the three-way ANOVAs (panelist ID, replicate, and product type as fixed effects + their first-order interactions) examining the effects of beverage type (all 12 beverages) on the time to the first instance of swallowing and the total duration of evaluation using the mono-intake TDS profiling data (total degrees of freedom: 359; residual degrees of freedom: 154). Significant *p*-values are in bold (α = 0.05).

	Panelist ID		Replicate		Product Type		Panelist ID * Replicate		Panelist ID * Product Type		Replicate * Product Type	
	*F*	*p-*Value	*F*	*p-*Value	*F*	*p-*Value	*F*	*p-*Value	*F*	*p-*Value	*F*	*p-*Value
Time to first swallow	35.82	**<0.01**	0.01	0.90	3.43	**0.00**	0.46	0.95	1.40	**0.02**	0.71	0.73
Total duration of evaluation	158.70	**<0.01**	1.03	0.31	6.51	**<0.01**	0.84	0.63	1.98	**<0.01**	2.31	**0.01**

* corresponds to the interaction between replicate and product type.

**Table 6:** Results of the four-way ANOVAs (panelist ID, product type, spoonful ID, and replicate as fixed effects + their first-order interactions) examining the effects of beverage type (only I/F+/G+/S− and P/F+/G+/S−) on the time to the first instance of swallowing and the total duration of evaluation using the multi-intake TDS profiling data (total degrees of freedom: 191; residual degrees of freedom: 107). Significant *p*-values are in bold (α = 0.05). Abbreviations: I = isolate, P = pellet, F+ = 1.5% oil, G+ = 0.5% gellan gum, and S− = 0.08% salt.

	Panelist ID		Product Type		Spoonful ID		Replicate		Panelist ID * Product Type		Panelist ID * Spoonful ID		Panelist ID * Replicate		Product Type * Spoonful ID		Product Type * Replicate		Spoonful ID * Replicate	
	*F*	*p-*Value	*F*	*p-*Value	*F*	*p-*Value	*F*	*p-*Value	*F*	*p-*Value	*F*	*p-*Value	*F*	*p-*Value	*F*	*p-*Value	*F*	*p-*Value	*F*	*p-*Value
Time to first swallow	33.07	**<0.01**	4.70	0.03	11.48	**<0.01**	0.06	0.81	4.29	**<0.01**	0.85	0.68	2.98	**<0.01**	1.94	0.15	1.06	0.31	1.24	0.29
Total duration of evaluation	61.87	**<0.01**	2.74	0.10	7.10	**<0.01**	10.54	0.00	2.15	0.01	1.81	0.02	3.42	**<0.01**	0.76	0.47	0.10	0.76	0.26	0.77

* corresponds to the interaction between replicate and product type.
